# Elevated extracellular water to total body water ratio and low phase angle in relation to muscle function in middle-aged and older adults

**DOI:** 10.1080/15502783.2025.2536693

**Published:** 2025-08-07

**Authors:** Ting-Fu Lai, Jong-Hwan Park, Minwoo Jang, Jiaren Chen, Myung-Jun Shin, Eunsoo Moon, Jung Mo Kang, Jong Won Lee, Yoon Jae Cho, Yung Liao, Tae Sik Goh, Jung Sub Lee

**Affiliations:** a Pusan National University Hospital, Biomedical Research Institute, Busan, Republic of Korea; b National Taiwan Normal University, Graduate Institute of Sport, Leisure and Hospitality Management, Taipei City, Taiwan; c Pusan National University School of Medicine, Department of Convergence Medicine, Yangsan, Republic of Korea; d Pusan National University School of Medicine, Department of Clinical Bio-Convergence, Graduate School of Convergence in Biomedical Science, Yangsan, Republic of Korea; e Pusan National University Hospital, Convergence Medical Institute of Technology, Busan, Republic of Korea; f Pusan National University School of Medicine, Department of Rehabilitation Medicine, Yangsan, Republic of Korea; g Pusan National University Hospital, Department of Rehabilitation Medicine, Busan, Republic of Korea; h Pusan National University School of Medicine, Department of Psychiatry, Yangsan, Republic of Korea; i Pusan National University Hospital, Department of Psychiatry , Busan, Republic of Korea; j Pusan National University School of Medicine, Department of Orthopaedic Surgery,Yangsan, Republic of Korea; k Pusan National University Hospital, Department of Orthopaedic Surgery, Busan, Republic of Korea; l Faculty of Sport Sciences, Waseda University, Tokorozawa, Japan

**Keywords:** Phase angle, ECW/TBW, muscle function, grip strength, physical performance, bioelectrical impedance analysis

## Abstract

**Background:**

Decline in muscle function is a major health concern in aging populations, increasing the risk for disability and frailty. Bioelectrical impedance analysis (BIA) offers a practical method to assess physiological status in community settings. BIA-derived parameters include the phase angle (PhA), an indicator of cellular health and integrity, and the extracellular water to total body water (ECW/TBW) ratio, which reflects fluid balance and systemic inflammation. Objective: While these markers have been independently linked to adverse health outcomes, their combined utility for stratifying the risk of functional decline remains underexplored. This study aimed to investigate the joint association of PhA and ECW/TBW status with poor muscle function in middle-aged and older adults.

**Methods:**

This cross-sectional study included 695 community-dwelling adults aged ≥ 50 years (mean age 75.7 ± 8.9 years; 21.4% male; mean BMI 24.6 ± 3.6 kg/m^2^). PhA and ECW/TBW were measured using a BWA 2.0 Body Water Analyzer (InBody BWA, Inc. Audubon, PA, USA) in a seated position. Participants were categorized into three groups based on their PhA and ECW/TBW status: Normal PhA/Normal ECW/TBW (*n* = 258), Normal PhA/Elevated ECW/TBW (*n* = 323), and Lower PhA/Elevated ECW/TBW (*n* = 114). Logistic regression was used to assess the association with low physical function (Short Physical Performance Battery score ≤ 9) and low grip strength.

**Results:**

After adjustment for demographic and clinical confounders, the Lower PhA/Elevated ECW/TBW group showed significant associations with both low physical function (OR = 3.07, 95% CI = 1.63–5.81) and low grip strength (OR = 2.41, 95% CI = 1.20–4.85), as well as their co-occurrence (OR = 3.10, 95% CI = 1.53–6.27). No significant associations were found for the Normal PhA/Elevated ECW/TBW group after adjustment.

**Conclusion:**

The combination of a lower PhA and an elevated ECW/TBW ratio is significantly associated with poor muscle function. This combined BIA profile may serve as a useful, noninvasive screening tool for identifying individuals at high risk of functional decline in community settings.

## 1. Introduction

Maintaining muscle function is crucial for overall health and well-being, particularly in older adults. Age-related decline in muscle function, often termed dynapenia and sarcopenia [1,2], is associated with increased risk of falls, frailty, disability, and reduced quality of life [3,4]. Therefore, identifying individuals at risk of muscle dysfunction or mobility limitation is a critical public health priority.

Bioelectrical impedance analysis (BIA) is a promising technique for assessing body composition and physiological status [5]. Two key parameters derived from BIA, phase angle (PhA) and the extracellular water-to-total body water (ECW/TBW) ratio, have been independently linked to muscle health. Specifically, a lower PhA, considered a proxy for compromised cellular membrane integrity, has been shown to be independently associated with decreased grip strength, slower gait speed, and poorer overall physical performance in older adults [6–8]. Similarly, an elevated ECW/TBW ratio, which may indicate systemic inflammation or fluid imbalance, has also been associated with functional decline and an increased risk for sarcopenia [9–11].

However, these two markers reflect distinct, albeit related, physiological processes: PhA provides insight into cellular quality and integrity, while the ECW/TBW ratio relates more to fluid distribution and the extracellular environment. Prior research has often examined these markers in isolation or only within populations already diagnosed with sarcopenia [12–14]. Consequently, the potential for their combined use to offer superior risk stratification for functional decline in a broader population of middle-aged and older adults remains underexplored. We hypothesized that combining these markers could provide a more nuanced assessment. For instance, an individual might present with only one abnormal marker (e.g. inflammation-driven fluid shifts but intact cellular health), representing an intermediate risk. In contrast, the simultaneous presence of both a low PhA and an elevated ECW/TBW ratio could signify a synergistic deterioration of both cellular quality and its surrounding environment, thereby identifying a subgroup with the highest risk of functional impairment.

Therefore, this study aimed to investigate the relationship between the combined presence of elevated ECW/TBW ratio and low PhA and muscle function in middle-aged and older adults. We hypothesized that individuals exhibiting both an elevated ECW/TBW ratio and a low PhA would demonstrate poorer muscle function compared to those with only one or neither of these unfavorable markers. By clarifying this relationship, this study contributes to a better understanding of the association between fluid distribution, cellular health, and muscle function, potentially informing the development of more effective strategies for maintaining muscle health in aging populations.

## 2. Methods

### 2.1. Participants

**Figure 1.**
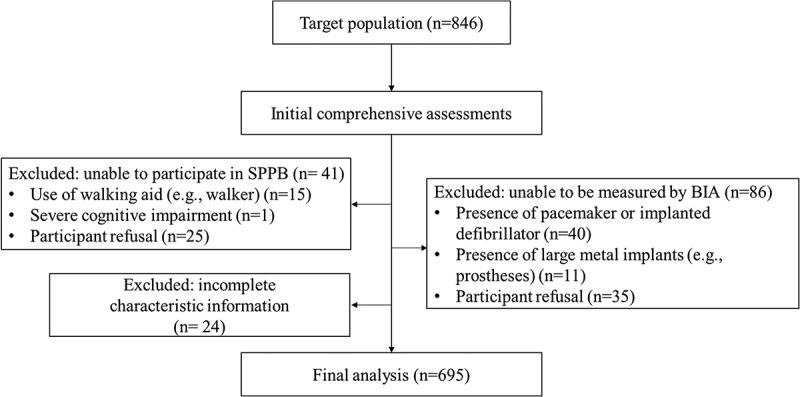
Participant flowchart.

Participants were recruited from medically vulnerable adult community groups residing in Busan, South Korea, as part of the 2023 BUSAN Screening and Assessment Network study at Pusan National University Hospital. This cross-sectional study was conducted from March 2023 to November 2023. The target population consisted of individuals aged ≥50 years who fell into one or more of the following categories: low income, living in single-person households, diagnosed with chronic conditions, or living alone. Additional medically vulnerable groups were also considered eligible to participate. A total of 846 participants were included in this study. The Busan Metropolitan City Government pre-screened the target population to identify potential participants who met the inclusion criteria. The medical staff then visited selected individuals at their residences to perform comprehensive health assessments, including individual or family history of chronic diseases, basic anthropometric measurements, body composition, hand-grip strength, and the Short Physical Performance Battery (SPPB). From this initial group, participants were excluded if they were unable to complete the SPPB (*n* = 41), primarily due to the use of a walking aid or severe cognitive impairment; had contraindications for BIA measurement (*n* = 86), such as the presence of a pacemaker or large metal implants; or had incomplete characteristic information (*n* = 24). This resulted in a final sample of 695 participants for analysis. The participant selection process is detailed in Figure 1. This study was conducted in accordance with the Declaration of Helsinki and approved by the Institutional Review Board of the Pusan National University Hospital (IRB number 2206–016–116).

### 2.2. Measurements

#### 2.2.1. Bioelectrical impedance analysis

Body composition was assessed using a direct segmental multifrequency bioelectrical impedance analysis (DSM-BIA) device, the BWA 2.0 Body Water Analyzer (InBody BWA, Inc., Audubon, PA, USA). This device uses an eight-point tactile electrode system with frequencies ranging from 1 kHz to 3000 kHz (3 MHz) to measure segmental impedance. All assessments were performed according to a standardized protocol [15]. Participants were measured in a seated position after a minimum 10-minute rest period. As per standard BIA procedure, clamp-type electrodes were attached to the participant’s ankles and wrists. The skin at the electrode sites was cleaned with an alcohol swab prior to placement to ensure proper contact. To minimize the effects of diurnal fluid shifts, all assessments were conducted during the morning hours (8:00 AM–11:00 AM). From this assessment, two key parameters were used for analysis. The whole-body phase angle (PhA) at 50 kHz was used as an indicator of cellular health and integrity [15,16], with lower PhA defined by cutoffs of <5.04° for men and <4.20° for women [17]. The validity of using high-frequency, segmental BIA for assessing body composition has been established against reference methods. Previous studies have demonstrated its accuracy for estimating parameters such as lean soft tissue mass [16]. Furthermore, and of particular relevance to sarcopenia research, a recent study by Yi et al. (2022) confirmed that the BWA device shows excellent agreement with DEXA for the assessment of appendicular lean mass, reporting a high correlation (standardized β ≥ 0.95) in adults [17].

##### 2.2.1.1. ECW/TBW ratio

The Extracellular Water to Total Body Water (ECW/TBW) ratio was used to assess body fluid distribution. This ratio was not calculated manually but was obtained directly from the output of the InBody BWA 2.0 device. The device estimates ECW and TBW values using a proprietary algorithm developed by the manufacturer, which is based on the multi-frequency impedance measurements. For the purpose of analysis, participants with an ECW/TBW ratio higher than 0.390 were categorized as having an elevated ratio [18].

#### 2.2.2. SPPB

The SPPB is an objective assessment tool for evaluating physical function and mobility in older adults. It consists of balance, gait speed, and chair-stand tests [19,20]. Balance tests were performed in three progressively more challenging standing positions: feet together, semi-tandem, and tandem stance. Each position was maintained for 10 s. The gait speed test was used to measure the time required to walk a distance of 4 m at a normal pace. The chair-stand tests were implemented by asking the participants to assume a seated position five times in the standing position with their arms folded across the chest. The score was 0–4 for each test, leading to a total score of 0–12, with higher scores indicating a better performance [21]. In this study, a score of ≤ 9 was used to define low physical function. This cut-point is based on the seminal work of Guralnik et al. that first demonstrated the SPPB’s predictive validity for future disability, and is further supported by large-scale meta-analyses linking lower scores to increased all-cause mortality [20,22].

#### 2.2.3. Hand-grip strength

Hand-grip strength was measured using a Digital Grip Strength Dynamometer TKK 5401 (Takei Scientific Instruments Co. Ltd., Tokyo, Japan), which has a reported accuracy of ±2 kg and a resolution of 0.1 kg [23]. Each participant made two attempts to measure the dominant/non-dominant hand-grip strength, and the dynamometer showed the highest performance of the two attempts. During the assessment, participants were instructed to be in a seated position, with their shoulder adducted and neutrally rotated, elbow flexed at 90 degrees, and the wrist in a neutral position. Measurements were performed on both dominant and non-dominant hands. Two trials were conducted for each hand, with a rest period of approximately 60 seconds between each attempt to prevent fatigue. For the final analysis, the maximum value recorded from all trials, regardless of the hand used, was taken as the participant’s handgrip strength score.

#### 2.2.4. Covariates

Covariates were selected based on their potential influence on the primary outcomes. These included data obtained from BIA measurements, physical measurements, and an interviewer-administered questionnaire. BMI and percentage of body fat were obtained directly from the BIA device results. Abdominal circumference was measured manually on-site by trained staff using a non-stretchable tape measure at the approximate midpoint between the lowest rib and the iliac crest, following standardized procedures. Demographic information, including age and sex, as well as data on health history and behaviors, were collected via a structured questionnaire administered by a trained interviewer. This included alcohol use, history of stroke treatment, smoking status (categorized as never smoked, former smoker, or current smoker), presence of neuropsychiatric problems, and a history of physician-diagnosed comorbidities such as diabetes, hypertension, and cancer. Finally, the time spent in moderate-to-vigorous physical activity (MVPA) per week was assessed using the validated Korean Global Physical Activity Questionnaire [24].

### 2.3. Statistical analyses

Participant characteristics were analyzed using IBM SPSS Statistics (version 27.0; IBM Corp., New York, USA). Based on the pre-defined cutoff values for PhA and the ECW/TBW ratio (defined as PhA < 5.04° for men and < 4.20° for women, and ECW/TBW ratio > 0.390), participants were stratified based on four potential combinations. However, we observed that the “Lower PhA × Normal ECW/TBW” group was naturally absent (*n* = 0), as every participant with a lower PhA also exhibited an elevated ECW/TBW ratio (Table 1). Consequently, for the purpose of analysis, participants were categorized into the following three observed groups: (1) Normal PhA × Normal ECW/TBW, (2) Normal PhA × Elevated ECW/TBW, and (3) Lower PhA × Elevated ECW/TBW. This grouping strategy allowed us to investigate the combined and interactive effects of PhA and ECW/TBW on muscle function. Descriptive statistics were used to summarize participant characteristics within each group. Continuous variables are presented as means ± standard deviations (SD), and categorical variables are presented as frequencies and percentages. To compare continuous variables across the three groups, the assumption of homogeneity of variances was first assessed using Levene’s test. Standard one-way ANOVA was used for variables that met the assumption, while the more robust Welch’s ANOVA, which does not assume equal variances, was applied to variables that violated the assumption (*p* < 0.05). Chi-square tests were used to compare categorical variables. To investigate the association of the PhA – ECW/TBW groups with muscle function, binary logistic regression analyses were performed, with the Normal PhA × Normal ECW/TBW group serving as the reference. The dependent variables were: (1) low physical function (SPPB total score ≤ 9), (2) low handgrip strength (not meeting the cut-offs of ≥28 kg for men and ≥18 kg for women, based on the Asian Working Group for Sarcopenia (AWGS) consensus [2]), and (3) concurrently having both low physical function and low handgrip strength. A p-value < 0.05 was considered statistically significant.

**Table 1.** Distribution of participants by phase angle and ECW/TBW ratio categories.

	ECW/TBW Ratio		
Phase Angle (PhA) Group	Normal	Elevated	Total
Normal PhA	258 (100.0%)	323 (77.9%)	581 (85.6%)
Lower PhA	0 (0.0%)	114 (22.1%)	114 (14.4%)
Total	258 (100.0%)	437 (100.0%)	695 (100.0%)

Data are presented as n (% within column). PhA = Phase Angle; ECW/TBW = Extracellular Water to Total Body Water Ratio.

## 3. Results

### 3.1. Characteristics of the participants

A total of 695 participants with complete data were included in the final analysis. For the primary analysis, participants were stratified into four potential groups based on their PhA and ECW/TBW status. The distribution of participants across these categories is presented in Table 2. Notably, a strong co-occurrence was observed between the two BIA-derived parameters, as no participants were found in the “Lower PhA and Normal ECW/TBW” category (*n* = 0). This resulted in three distinct groups for subsequent analysis: Normal PhA × Normal ECW/TBW (*n* = 258), Normal PhA × Elevated ECW/TBW (*n* = 323), and Lower PhA × Elevated ECW/TBW (*n* = 114). The demographic and clinical characteristics of these three groups are compared in Table 2. Significant differences were observed across the groups in several characteristics. For example, the Lower PhA × Elevated ECW/TBW Ratio group was characterized by the highest mean age (81.91 ± 7.42 years), which was significantly higher than both the Normal PhA × Normal ECW/TBW Ratio (69.33 ± 8.12 years) and Normal PhA × Elevated ECW/TBW Ratio groups (78.64 ± 6.62 years) (*p* < 0.001). Gender distribution also varied significantly among groups (*p* < 0.001), with men comprising 30.6%, 9.3%, and 35.1% of the Normal PhA×Normal ECW/TBW Ratio, Normal PhA×Elevated ECW/TBW Ratio, and Lower PhA×Elevated ECW/TBW Ratio groups, respectively. The Lower PhA×Elevated ECW/TBW Ratio group showed the lowest BMI (23.34 ± 3.59 kg/m^2^) compared to the other two groups (*p* < 0.001). Physical activity levels, measured as MVPA per week, were highest in the Normal PhA×Normal ECW/TBW Ratio group (234.32 ± 257.24 minutes) and progressively decreased in the other groups (*p* < 0.001). Regarding health behaviors and comorbidities, alcohol use was more prevalent in the Normal PhA×Normal ECW/TBW Ratio group (27.1%) compared to the other groups (*p* < 0.001). Hypertension showed an increasing trend across groups, being highest in the Lower PhA×Elevated ECW/TBW Ratio group (68.4%, *p* < 0.001). Notably, neuropsychiatric problems were more prevalent in the Lower PhA×Elevated ECW/TBW Ratio group, with 18.4% having severe dementia or depression compared to 12.0% and 9.6% in the other groups (*p* = 0.019). The unadjusted cross-tabulated distribution of participants by their Phase Angle group and SPPB performance, a key functional outcome, is provided in Supplementary Table S1.

**Table 2.** Characteristics of participants (*n* = 695).

Categorical variables	Normal PhA×Normal ECW/TBW Ratio (*n* = 258)	Normal PhA×Elevated ECW/TBW Ratio (*n* = 323)	Lower PhA×Elevated ECW/TBW Ratio (*n* = 114)	p-value
Gender, men (%)	79 (30.6)	30 (9.3)	40 (35.1)	<0.001
Alcohol use, yes (%)	70 (27.1)	47 (14.6)	16 (14.0)	<0.001
Stroke Treatment History, yes (%)	22 (8.5)	25 (7.7)	9 (7.9)	0.939
Smoking status (%)				<0.001
Never smoked	186 (72.1)	290 (89.8)	80 (70.2)	
Current smoker	26 (10.1)	11 (3.4)	9 (7.9)	
Former smoker	46 (17.8)	22 (6.8)	25 (21.9)	
Neuro psychiatric problems (%)				0.019
Severe dementia or depression	31 (12.0)	31 (9.6)	21 (18.4)	
Mild dementia	9 (3.5)	23 (7.1)	10 (8.8)	
None/No symptoms	218 (84.5)	269 (83.3)	83 (72.8)	
History of diabetes diagnosis, yes (%)	68 (26.4)	89 (27.6)	43 (37.7)	0.067
History of hypertension diagnosis, yes (%)	121 (46.9)	199 (61.6)	78 (68.4)	<0.001
History of other cancer diagnosis, yes (%)	29 (11.2)	35 (10.8)	12 (10.5)	0.977
History of dyslipidemia diagnosis, yes (%)	120 (46.5)	121 (37.5)	41 (36.0)	0.048
Continuous variables				
Body Mass Index, mean (SD); kg/m^2^	24.79 (3.73)^c^	24.94 (3.50)^c^	23.34 (3.59)^b^	<0.001
Age, mean (SD); years	69.33 (8.12)^b^	78.64 (6.62)^c^	81.91 (7.42)^d^	<0.001^a^
Circumference of Abdomen, mean (SD); cm	81.70 (9.53)	80.94 (8.16)	79.52 (9.44)	0.093
Percentage of body fat (SD); %	31.07 (6.98)^b^	34.63 (6.15)^c^	32.85 (7.82)^b^	<0.001^a^
MVPA per week, mean (SD); minute	234.32 (257.24)^c^	175.82 (230.60)^b^	133.48 (228.59)^b^	<0.001
Phase angle, mean (SD); degree	5.95 (0.61)^d^	4.93 (0.40)^c^	3.99 (0.45)^b^	<0.001^a^
ECW/TBW ratio, mean (SD)	0.385 (0.00)^b^	0.396 (0.00)^c^	0.404 (0.01)^d^	< 0.001^a^
Chair stand test (SD); sec	9.86 (4.09)^b^	10.61 (4.28)^b^	12.06 (5.33)^c^	0.001
Four-meter gait speed (SD); m/s	1.04 (0.36)^c^	0.98 (0.32)^c^	0.87 (0.56)^b^	0.001

Data are presented as mean ± SD for continuous variables and n (%) for categorical variables. P-values were derived from the Chi-square test for categorical variables.

^a^P-value obtained from Welch’s ANOVA due to heterogeneity of variances; other p-values for continuous variables were from standard one-way ANOVA.

^b,c,d^Within rows for continuous variables, means not sharing a common superscript letter are significantly different (*p* < 0.05) based on post-hoc tests with Sidak correction.

Abbreviations: SD, standard deviation; PhA, phase angle; ECW/TBW, extracellular water to total body water ratio; BMI, body mass index; MVPA, moderate-to-vigorous physical activity.

### 3.2. Joint associations of lower PhA and elevated ECW/TBW ratios with low physical function and low grip strength as separate outcomes

Table 3 presents the joint associations of PhA and ECW/TBW ratio status with both mobility limitation and grip strength. In the unadjusted model, compared to the reference group (Normal PhA × Normal ECW/TBW), participants with Normal PhA × Elevated ECW/TBW had significantly elevated odds of mobility limitation (OR = 1.73, 95% CI = 1.13–2.65) and lower grip strength (OR = 1.85, 95% CI = 1.33–2.58). The Lower PhA × Elevated ECW/TBW group exhibited even stronger associations with both mobility limitation (OR = 6.46, 95% CI = 3.91–10.67) and lower grip strength (OR = 5.84, 95% CI = 3.37–10.12). After adjusting for demographic variables, lifestyle factors, and comorbidities, the associations for the Normal PhA × Elevated ECW/TBW group were no longer statistically significant for either low physical function (OR = 1.01, 95% CI = 0.60–1.70) or grip strength (OR = 0.79, 95% CI = 0.51–1.22). However, the Lower PhA × Elevated ECW/TBW group retained significant associations with both low physical function (OR = 3.07, 95% CI = 1.63–5.81) and low grip strength (OR = 2.41, 95% CI = 1.20–4.85).

**Table 3.** Joint associations of lower PhA and elevated ECW/TBW ratios with low physical function and low grip strength as separate outcomes.

	Low physical function				Low grip strength			
	Unadjusted model		Adjusted model^a^		Unadjusted model		Adjusted model^a^	
	OR (95% CI)	*p*-value	OR (95% CI)	*p*-value	OR (95% CI)	*p*-value	OR (95% CI)	*p*-value
Normal PhA×Normal ECW/TBW Ratio (*n* = 258)	1.00 (ref.)		1.00 (ref.)		1.00 (ref.)		1.00 (ref.)	
Normal PhA×Elevated ECW/TBW Ratio (*n* = 323)	1.73 (1.13–2.65)	0.012	1.01 (0.60–1.70)	0.969	1.85 (1.33–2.58)	< 0.001	0.79 (0.51–1.22)	0.292
Lower PhA×Elevated ECW/TBW Ratio (*n* = 114)	6.46 (3.91–10.67)	< 0.001	3.07 (1.63–5.81)	< 0.001	5.84 (3.37–10.12)	< 0.001	2.41 (1.20–4.85)	0.013

^a^Adjusted for demographic variables, including age, gender, BMI, abdominal circumference, percentage of body fat, alcohol use, history of stroke treatment, smoking status, presence of neuropsychiatric problems, previously diagnosed comorbidities such as diabetes, hypertension, and cancer, and time spent in MVPA.

Diagnosed comorbidities such as diabetes, hypertension, and cancer, and time spent in MVPA.

### 3.3. Joint associations of lower PhA and elevated ECW/TBW ratios with co-occurrence of low physical function and low grip strength

Table 4 presents the joint associations of PhA and ECW/TBW ratio status with the co-occurrence of low physical function and low grip strength. In the unadjusted model, compared to the reference group, both the Normal PhA × Elevated ECW/TBW group (OR = 2.60, 95% CI = 1.58–4.28) and the Lower PhA × Elevated ECW/TBW group (OR = 9.09, 95% CI = 5.20–15.88) had significantly increased odds of experiencing both conditions. After adjusting for the same covariates, the association for the Normal PhA × Elevated ECW/TBW group was no longer statistically significant (OR = 1.21, 95% CI = 0.67–2.19). However, the Lower PhA × Elevated ECW/TBW group maintained a strong and significant association with the combined outcome (OR = 3.10, 95% CI = 1.53–6.27).

**Table 4.** Joint associations of lower PhA and elevated ECW/TBW ratios with co-occurrence of low physical function and low grip strength.

	With co-occurrence of low physical function and low grip strength			
	Unadjusted model		Adjusted model^a^	
	OR (95% CI)	*p*-value	OR (95% CI)	*p*-value
Normal PhA×Normal ECW/TBW Ratio (*n* = 258)	1.00 (ref.)		1.00 (ref.)	
Normal PhA×Elevated ECW/TBW Ratio (*n* = 323)	2.60 (1.58–4.28)	< 0.001	1.21 (0.67–2.19)	0.518
Lower PhA×Elevated ECW/TBW Ratio (*n* = 114)	9.09 (5.20–15.88)	< 0.001	3.10 (1.53–6.27)	0.002

^a^Adjusted for demographic variables, including age, sex, BMI, abdominal circumference, percentage of body fat, alcohol use, history of stroke treatment, smoking status, presence of neuropsychiatric problems, previously diagnosed comorbidities such as diabetes, hypertension, and cancer, and time spent in MVPA.

## 4. Discussion

This study investigated the joint associations of PhA and ECW/TBW ratio with low physical function, grip strength, and their co-occurrence in adults aged 50 and above. Our findings revealed that the combination of lower PhA and elevated ECW/TBW was particularly strongly associated with both low physical function and reduced grip strength, and even more so with the presence of both limitations. This highlights the potential of combining PhA and ECW/TBW assessments to identify individuals at elevated risk of functional decline.

Our results indicate that lower PhA, particularly in combination with an elevated ECW/TBW ratio, is associated with low physical function and reduced grip strength in older adults. While both lower PhA and elevated ECW/TBW, when considered independently in unadjusted models, showed associations with these functional limitations, the combination of lower PhA and elevated ECW/TBW consistently demonstrated the associations, even after adjusting for various confounding factors. This suggests that the combined assessment of PhA and ECW/TBW provides more comprehensive information about functional decline risk than either measure alone. The association between PhA and functional limitations may be explained by its reflection of cellular health, integrity, and hydration status. Lower PhA values may indicate compromised cellular function, potentially reflecting reduced muscle quality and functional capacity. Conversely, higher PhA values are generally associated with better cellular function and overall health and have been used to assess nutritional status, muscle mass, and function [13]. These findings are consistent with previous research suggesting PhA’s sensitivity to changes in body composition related to muscle health [25]. However, it is important to note that our study focused on functional outcomes (lower body strength and grip strength) rather than a direct clinical diagnosis of sarcopenia as defined by specific diagnostic criteria (e.g. AWGS). The weaker association observed for elevated ECW/TBW alone in the adjusted models suggests that this measure may be less specific to muscle function and more influenced by fluctuations in body water compartments. While some studies have reported associations between ECW/TBW and sarcopenia [12], differences in study populations (e.g. age range) and the specific definition of sarcopenia used may contribute to the discrepancies. For example, the study by Park, Lee [12] focused on a population aged 60 years or older, potentially including individuals with more advanced age-related changes. Furthermore, while some research suggests the importance of ECW/TBW in diagnosing sarcopenia to account for overhydration [26], our findings, which focused on functional measures, did not replicate these specific associations.

An important observation from this study was the absence of a “Lower PhA × Normal ECW/TBW” group (*n* = 0) in our cohort, which may point to the interconnected nature of these two parameters. This finding suggests that in our population, a state of compromised cellular integrity (low PhA) is often accompanied by dysregulated fluid balance (elevated ECW/TBW). This context helps explain why the combined effect of these two states was particularly pronounced for the co-occurrence of both low physical function and reduced grip strength. Such an interaction suggests that the concurrent impairment of cellular function and fluid dysregulation may exacerbate the risk of experiencing combined functional decline [27,28]. The synergistic negative effect likely arises from a vicious cycle of physiological insults. A low PhA indicates compromised cellular integrity, which impairs a muscle cell’s ability to maintain electrochemical gradients and generate force [29]. Concurrently, an elevated ECW/TBW ratio often reflects systemic inflammation [11], a state where pro-inflammatory cytokines promote muscle catabolism and increase fluid retention [30]. Oxidative stress serves as a shared underlying pathway that can exacerbate both cell membrane damage and the inflammatory response [31]. Thus, this combination likely represents a state of comprehensive physiological compromise that is linked to a greater functional deficit than either factor in isolation. In contrast to this synergistic effect, an interesting finding was the non-significant association with functional limitations in the “Normal PhA × High ECW/TBW” group after full adjustment. This may suggest that preserved cellular integrity, as reflected by a normal PhA, is a primary determinant of muscle function [8], potentially protecting the cell’s functional capacity against the systemic stress indicated by an elevated ECW/TBW ratio [27]. In this view, a low PhA may represent the critical threshold at which significant functional decline occurs. Alternatively, any true effect in this group may have been too subtle for our study to detect after adjusting for multiple confounders. Future research with larger samples is needed to clarify the independent contribution of an elevated ECW/TBW in individuals with preserved cellular integrity.

Our study population, adults aged 50 and above, represents a demographic at increased risk for age-related declines in muscle mass, strength, and physical function [32,33]. According to the AWGS 2019 consensus [2], sarcopenia is defined by low muscle mass combined with low muscle strength or performance. We propose that the unfavorable BIA profile of “Lower PhA × Elevated ECW/TBW” may serve as a practical proxy for this condition, as the underlying cellular compromise and fluid dysregulation are mechanistically linked to muscle atrophy. Therefore, using multifrequency BIA to identify this combined profile offers a noninvasive and readily applicable “first-step” screening method in community settings. Early identification of these at-risk individuals could signal the need for definitive assessment (e.g. with DXA) and facilitate timely interventions, such as nutritional optimization and resistance exercise. Such strategies would be aimed at mitigating the progression of not just functional decline, but sarcopenia itself, thereby reducing the risk of associated adverse outcomes like falls, disability, and reduced quality of life. Future research should formally evaluate the diagnostic accuracy of this BIA profile against the full AWGS 2019 criteria.

### 4.1. Limitations

Some limitations should be acknowledged. First, body composition parameters were derived from manufacturer’s prediction equations rather than direct measurements like DXA or MRI. While these equations have been validated in Asian populations, this indirect measurement may introduce some uncertainty. It is important to distinguish that while the ECW/TBW ratio is an estimated value derived from these equations, the PhA is a raw parameter directly calculated from the measured resistance and reactance, reflecting the physical properties of the tissues. Future studies incorporating multiple measurement techniques would strengthen these findings. Second, our BIA measurement protocol has specific limitations. The use of device-specific reference values for PhA and ECW/TBW limits direct comparisons with studies using different BIA systems. This is because normative data and clinical cutoffs can vary significantly depending on the specific device, the prediction equations utilized, and the demographic characteristics of the reference population, necessitating caution when comparing absolute values across studies. Furthermore, while assessments were consistently conducted in the morning to minimize diurnal variation, other pre-assessment conditions were not fully standardized. Specifically, participants were not required to observe a strict fasting period or abstain from caffeine and exercise before measurement. These factors are known to influence body fluid status and could have introduced random variability into the BIA measurements [34]. However, as these conditions were unlikely to differ systematically across our analytical groups, such random error would typically bias our results toward the null, suggesting that the strong associations we observed are likely robust. Third, this study did not account for several potentially significant confounders. Specifically, detailed assessments of nutritional status (e.g. dietary protein intake), key hormonal profiles (e.g. testosterone, cortisol), and specific inflammatory markers (e.g. CRP, IL-6) were not performed [35]. Since these factors are known to be on the causal pathway of age-related functional decline and could simultaneously influence both BIA parameters and our outcomes, their absence may have confounded the observed associations. Future longitudinal studies should incorporate these measures to better elucidate the underlying mechanisms.

## 5. Conclusions

In conclusion, our study establishes that the combined BIA profile of a low PhA and an elevated ECW/TBW ratio serves as a noninvasive marker for identifying functional decline in middle-aged and older adults. Its application in community-based screenings could enable the early detection of at-risk individuals, creating a critical opportunity for targeted interventions, such as nutritional counseling and resistance exercise, to mitigate the progression toward sarcopenia and severe disability. Building on these findings, future longitudinal research is essential to determine if this BIA profile can also prospectively predict functional decline. Furthermore, studies incorporating key inflammatory and hormonal biomarkers are needed to fully elucidate the underlying biological mechanisms driving this association. Effectively integrating such practical biomarkers into public health strategies would be a step toward promoting healthy aging and preserving functional independence.

## Supplementary Material

Supplemental Material

## Data Availability

Data are available from the corresponding author upon reasonable request

## List of abbreviations

BIA: Bioelectrical impedance analysis
PhA: Phase angle
ECW/TBW: Extracellular water-to-total body water ratio
BMI: Body mass index
OR: Odd ratio
CI: confidence interval
MVPA: moderate-to-vigorous physical activity
BUSAN: BUs-based screening and assessment network study
SMI: Skeletal muscle mass index
SPPB: Short physical performance battery
