# Supplementary material for: Elevated extracellular water to total body water ratio and low phase angle in relation to muscle function in middle-aged and older adults
Source: J Int Soc Sports Nutr. 2025 Aug 7;22(1):2536693. doi: 10.1080/15502783.2025.2536693 (PMC12332989; doi:10.1080/15502783.2025.2536693)
Supplement: Supplemental Material [file RSSN_A_2536693_SM4485.docx]

Supplementary Table 1. Distribution of Participants by Phase Angle Group and SPPB Performance Group.

|  | SPPB Performance | |  |
| --- | --- | --- | --- |
| Phase Angle (PhA) Group | High Performance (>9) | Low Performance (≤9) | Total |
| Normal PhA | 466 (89.8%) | 115 (65.3%) | 581 (85.6%) |
| Lower PhA | 53 (10.2%) | 61 (34.7%) | 114 (14.4%) |
| Total | 519 (100.0%) | 176 (100.0%) | 695 (100.0%) |

Note. Data are presented as n (% within column).
